# Influence of mild-moderate hypocapnia on intracranial pressure slow waves activity in TBI

**DOI:** 10.1007/s00701-019-04118-6

**Published:** 2019-12-16

**Authors:** Erta Beqiri, Marek Czosnyka, Afroditi D. Lalou, Frederick A. Zeiler, Marta Fedriga, Luzius A. Steiner, Arturo Chieregato, Peter Smielewski

**Affiliations:** 1 Brain Physics Laboratory, Division of Neurosurgery, Department of Clinical Neurosciences, University of Cambridge, Cambridge, UK; 2 Department of Physiology and Transplantation, Milan University, Milan, Italy; 3 Department of Surgery, Rady Faculty of Health Sciences, University of Manitoba, Manitoba, Canada; 4 Division of Anaesthesia, Department of Medicine, University of Cambridge, Cambridge, UK; 5 Department of Anesthesia, Critical care and Emergency, Spedali Civili University Hospital, Piazzale Spedali civili 1, 25123 Brescia, Italy; 6 Anesthesiology, University Hospital Basel and Department of Clinical Research University of Basel, Basel, Switzerland; 7 Neurointensive Care Unit, Grande Ospedale Metropolitano Niguarda, Milan, Italy

**Keywords:** ICP slow waves, Hypocapnia, TBI, Cerebral autoregulation, Compensatory reserve, RAP

## Abstract

**Background:**

In traumatic brain injury (TBI) the patterns of intracranial pressure (ICP) waveforms may reflect pathological processes that ultimately lead to unfavorable outcome. In particular, ICP slow waves (sw) (0.005–0.05 Hz) magnitude and complexity have been shown to have positive association with favorable outcome. Mild-moderate hypocapnia is currently used for short periods to treat critical elevations in ICP. Our goals were to assess changes in the ICP sw activity occurring following sudden onset of mild-moderate hypocapnia and to examine the relationship between changes in ICP sw activity and other physiological variables during the hypocapnic challenge.

**Methods:**

ICP, arterial blood pressure (ABP), and bilateral middle cerebral artery blood flow velocity (FV), were prospectively collected in 29 adult severe TBI patients requiring ICP monitoring and mechanical ventilation in whom a minute volume ventilation increase (15–20% increase in respiratory minute volume) was performed as part of a clinical CO_2_-reactivity test. The time series were first treated using FFT filter (pass-band set to 0.005–0.05 Hz). Power spectral density analysis was performed. We calculated the following: mean value, standard deviation, variance and coefficient of variation in the time domain; total power and frequency centroid in the frequency domain; cerebrospinal compliance (Ci) and compensatory reserve index (RAP).

**Results:**

Hypocapnia led to a decrease in power and increase in frequency centroid and entropy of slow waves in ICP and FV (not ABP). In a multiple linear regression model, RAP at the baseline was the strongest predictor for the decrease in the power of ICP slow waves (*p* < 0.001).

**Conclusion:**

In severe TBI patients, a sudden mild-moderate hypocapnia induces a decrease in mean ICP and FV, but also in slow waves power of both signals. At the same time, it increases their higher frequency content and their morphological complexity. The difference in power of the ICP slow waves between the baseline and the hypocapnia period depends on the baseline cerebrospinal compensatory reserve as measured by RAP.

## Introduction

In traumatic brain injury (TBI) the mean value of intracranial pressure (ICP) might not be sufficient to help fully interpret the clinical status of the patient, while ICP waveforms contain information about the nature of the cerebrospinal circulation pathophysiology. ICP waveform can be decomposed into the following components defined in the frequency domain: the pulse waveform (which fundamental harmonic component frequency equals the heart rate), the respiratory waveform (related to the frequency of the respiratory cycle, 8–20 cycles/minute) and the “Slow waves” [7] which were previously defined in the Lundberg thesis as B waves with “frequency 0.5–2/min with amplitude from discernibility to 20 mmHg” [24] and which definition was modified and adjusted in the latest years. Slow waves can be defined as oscillations in cerebral pressures and cerebral blood flow of duration longer than those of the respiratory origin with a spectral representation within the frequency limits defined roughly as 0.005–0.05 Hz [7]. Analysis of slow waves in ICP could provide information about cerebral blood flow (CBF) autoregulation [36], brain compliance [27, 24] and brainstem activity [37, 22, 15]. Moreover, various parameters derived from slow waves, in particular higher magnitude [2] and higher complexity [23], were shown to be associated with outcome after TBI.

The vasogenic nature of ICP slow waves [37, 1] is underlined by the fact that rhythmic changes in cerebral blood volume (CBV) are transmitted into the ICP waveform [27]. Rhythmic oscillations in diameter of cerebral vessels can be triggered by fluctuations in mean arterial blood pressure (ABP) and/or by local neurochemical mechanisms [20, 16]. These vascular changes are responsible for alterations in CBV and subsequently contribute to the oscillations observed in the cerebral blood flow velocity (FV) measured at the middle cerebral artery (MCA) [25]. CBV slow waves are ultimately transmitted into the ICP waveform [27]. The intracranial compliance modulates the transmission of the vasogenic waves. An increase in ICP slow wave amplitude may be indicative of an exhausted cerebrospinal compensatory reserve [39]. Slow wave magnitude has been shown to be suppressed by general anesthesia in awake versus sedated and ventilated TBI patients [21].

The intracranial blood pressure slow oscillations are likely modulated by the mean arterial blood pressure (ABP) and the sympathetic cervical system [17]. The relationship between ICP and ABP slow waves depends on the status of the dynamic autoregulation. With properly functioning CBF autoregulation, ICP slow waves are thought to result from the autoregulatory response to spontaneous fluctuations of cerebral perfusion pressure (CPP; where CPP = ABP − ICP) [30], and therefore can be used to gauge cerebral autoregulation quantitatively [9]. Failure of autoregulation modifies the relationship between ABP and ICP [36] in such a way that the response in ICP to the fluctuations in ABP becomes pressure passive and depends on the arterial bed compliance [14].

Changes in PaCO2 were suggested to be involved in the generation of the ICP slow waves, in the original Lundberg description of what he defined as B waves [24]. The arterial pCO_2_ fluctuations are not considered anymore the main generator of slow waves since they can be seen in ventilated patients, where pCO_2_ is actively stabilized and can be assumed to be kept constant. They are indeed considered potential modulators of the slow waves activity [31, 10] given their influence on the vascular resistance (metabolic regulation of CBF). A decrease in PaCO2 (inducing brain alkalosis) produces, with intact vascular reactivity, an acute vasoconstriction which leads to a reduction of CBF and CBV and ultimately a decrease in ICP [4], as a function of intracranial compliance [40]. Prolonged prophylactic deep hyperventilation was used in the past to prevent ICP hypertension, but it is no longer recommended given the lack of positive association with outcome and harmful effect of lowering CBF in the most vulnerable areas to ischemic levels[26, 6]. On the contrary, mild hypocapnia is considered safe from the hemodynamic point of view [32].

The behavior of ICP slow waves during hypocapnia in TBI patients has not been extensively studied yet, and it is uncertain whether there are significant changes in their activity and their patterns. Given that slow wave patterns are currently used to inform the treating clinicians about important physiological parameters (such as cerebral autoregulation, brain stem activity, and intracranial compliance) it is necessary to evaluate how a clinical intervention such as hypocapnia could influence the slow waves activity and therefore modify the assessment of the related physiology.

Therefore, we conducted this retrospective study, bearing in mind two main objectives:

1. To assess changes in the ICP slow waves activity occurring following sudden onset of mild-moderate hypocapnia, using a variety of approaches, both in time and frequency domain.
2. To examine the relationship between changes in ICP slow wave activity and other physiological variables during the hypocapnia period.

## Materials and methods

We present a retrospective analysis of waveform recordings of ICP, ABP, and bilateral MCA blood flow velocity (left - FVl, and right - FVr), prospectively collected during CO_2_-reactivity studies in adult (age > 16 years) severe TBI patients requiring ICP monitoring and mechanical ventilation admitted in the Neurocritical Care Unit (NCCU) at Addenbrooke’s Hospital, Cambridge, from March 2001 to February 2002. Reaching back to digital recordings from past studies was motivated by new findings regarding slow ICP waves and the role of autoregulation assessment [21, 3].

As part of the clinical protocol on NCCU, all patients underwent routine testing of CO_2_-reactivity to aid prognostic stratification. The collection of these data was prospectively considered by the multidisciplinary NCCU Users Group, and it was agreed that because assessment of CO_2_-reactivity was part of normal clinical management and since no patient confidentiality issues were involved, formal informed consent was not required. Within our institution, patient data may be collected with waiver of formal consent, as long as it remains fully anonymized, with no method of tracing this back to an individual patient. This anonymous data is then provided for future research purposes. Such data curation remains within compliance for research integrity as outlined in the UK Health Departments (2011) Governance arrangements for research ethics committees.

Exclusion criteria included respiratory failure, a baseline pa CO_2_ < 4.30 kPa, failure to obtain satisfactory bilateral transcranial Doppler signals and decompressive craniectomy. All patients were treated according to a CPP-orientated protocol aiming to keep CPP above 70 mmHg, ICP below 25 mmHg, and jugular bulb venous oxygen saturation (SjvO_2_) above 50%.

During the studies, all physiological parameters were maintained within the limits specified in the treatment guidelines of the unit. All patients were sedated with propofol (2–5 mg/kg/h) and fentanyl (1–2 mcg/kg/h), and paralyzed (atracurium). Infusion rates of sedative and vasoactive drugs were not changed and body temperature was kept constant throughout the study period.

### Data collection

The data were collected as part of a prospective study [35]. The setting up can be briefly described as follows.

ICP monitoring was performed using an intraparenchymal probe (Codman MicroSensors ICP Transducer, Codman & Shurtleff, Raynham, MA, USA). ABP was monitored invasively using a pressure monitoring kit (Baxter Healthcare CA, USA; Sidcup, UK) at the radial artery, zeroed at the level of the heart. Mainstream end-tidal CO_2_ monitoring was used (Marquette Solar 8000M, GE Medical Systems, UK) to assess the stability of CO_2_ levels, but the related signal was not collected. FV was measured from the middle cerebral arteries (left and right) with two 2-MHz probes with the Doppler Box (Multi Dop X4, DWL Elektronische Systeme, Sipplingen, Germany). The two probes were held in place with a Lam head rack. SjO_2_ was also monitored.

Data were collected during routine determination of CO_2_-reactivity as part of the standard clinical practice on the unit. After recording baseline data for 20 min and obtaining a baseline value for PaCO_2_ (AVLOmni, Roche Diagnostics GmbH, Graz, Austria), the minute volume of the ventilator was increased by a relative value of 15–20% of the original amount. If due to this intervention the unit’s standard treatment guidelines (PaCO_2_ > 3.5 kPa and or SjO_2_ > 55%) were exceeded, the protocol was abandoned. After an initial stabilization period of 10 min, end-tidal CO_2_ was kept stable and data were recorded for further 20 min. PaCO_2_ was measured at the middle of this stable phase (2 or 3 samples). During the study, all patients were sedated with propofol, (2–5 mg/kg/h) and fentanyl (1–2 mcg/kg/h), and paralyzed (atracurium). Infusion rates of sedative and vasoactive drugs were not changed and body temperature was kept constant throughout the study period. After CO_2_-reactivity testing had been completed, PaCO_2_ was slowly adjusted to the level that the responsible physician deemed appropriate.

The signals were acquired with a sampling frequency of 30 Hz using an analogue–digital converter (DT9801 and DT9803, Data Translation, Marlboro, MA, USA), and recorded using ICM+® software (Cambridge Enterprise, Cambridge, UK, http://icmplus.neurosurg.cam.ac.uk).

### Data processing

ICM+® software (Cambridge Enterprise Ltd, Cambridge, UK, http://icmplus.neurosurg.cam.ac.uk) was used to process the recorded signals.

The artifacts were manually removed in the 30 Hz raw data: in the ABP signal, the arterial line flushes (corresponding to the arterial blood sampling) were removed; in ABP, ICP, FVl, and FVr signal transient events, defined as short-lasting (less than 10 min) events occurring less frequently than 3/10 min, were removed to eliminate their influence on slow wave frequency bandwidth; any other artifacts found in recording were removed.

Per each patient recording, two periods—baseline and hypocapnic challenge—could be detected according to variations in the ICP trend (it decreases in hypocapnia; the initial transition and stabilization period after the increase in minute volume was excluded), the blood sampling time points (visible as arterial line artifact in the ABP signal) for the arterial pCO_2_ measurements, and the values of the reported pCO_2_ measurements (available in 26/29 patients).

#### Time trends and slow wave component

**Fig. 1:**
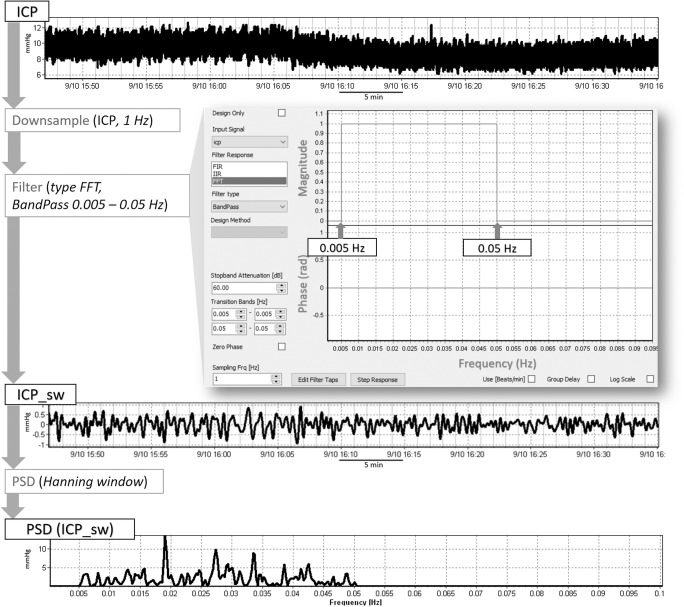
Signal processing methodology applied to obtain the slow wave component of the recorded signals. The whole pipeline is shown here for ICP. The raw waveform signals (time trend at the top) were first decimated to sampling frequency 1 Hz and subsequently processed with a FFT band-pass filter designed as shown in the picture, in order to isolate the slow wave component (0.005–0.05 Hz) of the four waveforms. Power spectral density (PSD) analysis was performed (periodogram method with the settings as shown in the picture)

In order to isolate the slow wave (sw) component (0.005–0.05 Hz) of the raw waveform signals, they were first decimated to sampling frequency 1 Hz and subsequently processed with an FFT band-pass filter. Four new signals were obtained as trends in the time domain: ABP_sw, ICP_sw, FVl_sw, and FVr_sw. In addition, power spectral density (PSD) [19] analysis was performed (periodogram method using the Hanning window) and results reported for the specified frequency range (Fig. 1). The PSD analysis allows estimation of the distribution of energy of the signal (power is energy expenditure over time) over frequency. In the context of slow waves, it simply provides a measure of variability of the analyzed signal over the specified frequency range and is calculated as an integral of the PSD function over the frequency range. For intuitive simplicity, if a square root was to be applied to the slow wave power estimations, this could be interpreted as an “equivalent amplitude” of a pure sinusoidal wave that would carry the same amount of energy. So in a sense, this parameter represents the amplitude of the slow waves.

Descriptive measures of the studied variables were extracted for each valid period per each patient. The periods were considered valid if they included at least 10 min of continuous data without gaps after the artifact removal and the filter application (with edge effects removed). The following metrics were calculated in the time domain: mean value, standard deviation, variance, coefficient of variation. To evaluate the complexity of the waveform, entropy was also calculated in the time domain (sample entropy, SaEn with length “m” = 2) [29]. Entropy is the rate of information production, a measurement of the system randomness or unpredictability. We investigated Entropy as a measure of the complexity of the time series.

In the frequency domain, limited to the studied range of frequencies, the following metrics were extracted: total power and frequency centroid. Given that the power of the slow waves is distributed in a range of frequencies rather than in one main frequency, we studied the frequency centroid as a measure of average frequency or a measure of the shape of the frequency distribution, and described its changes during hypocapnia.

In addition, ∆Power was calculated as Power_baseline_ − Power_hypocapnia_, ∆Centroid = Centroid_baseline_ − Centroid_hypocapnia_, and ∆Entropy = Entropy_baseline_ − Entropy_hypocapnia_.

#### Derived indexes

Coherence between FV and ICP was calculated as the maximum coherence in the frequency range 0.005–0.05 Hz between the two signals in time on the 30 Hz data.

We calculated cerebrospinal compliance (Ci) and the compensatory reserve index (RAP) to describe their influence in the modulation of the transmission of the vasogenic waves in the ICP waveform.

Ci was calculated as:

$$ \mathrm{Ci}=\frac{\mathrm{aCaBV}}{\mathrm{aICP}}\kern0.75em \left[\frac{\mathrm{c}{\mathrm{m}}^3}{\mathrm{m}\mathrm{mHg}}\ \right] $$

with aCaBV being the Fourier amplitude of the fundamental harmonic of the pulse of the cerebral arterial blood volume (CaBV). CaBV was derived from FV signal:

$$ \mathrm{CaBV}(t)=\int \left(\mathrm{FV}(t)-\mathrm{mean}\left(\mathrm{FV}\right)\right)\  dt\kern0.5em \left[\mathrm{c}{\mathrm{m}}^3\right] $$

where mean (FV) was calculated using a moving average filter (finite response filter) applied to FV [5]. ∆Ci = Ci_baseline_ − Ci_hypocapnia_.

RAP was calculated as the moving correlation coefficient between slow changes in ICP pulse amplitude (aICP = fundamental harmonic of the Fourier transformation of the pulse of ICP) and mean ICP (10 s average data) over a period of 5 min, updating every minute [8]. A Fisher transformation was separately applied to RAP (RAP_FT) for the purpose of the further statistical analysis.

The mean values of coherence, Ci and RAP, were extracted for period 1 (baseline) and for period 2 (hypocapnia).

### Statistical analysis

R statistical language was used to perform the statistical analysis [R: A language and environment for statistical computing. R Foundation for Statistical Computing, Vienna, Austria. URL http://www.R-project.org/. version 3.3.3]. The variables were summarized as mean values ± SD during the baseline and the hypocapnia period. The effects of hypocapnia in the exported parameters were studied using univariate tests (paired *t* test) comparing baseline vs hypocapnia. The correlations were performed with the Pearson method. For all tests, alpha was set at 0.05 for significance. Univariate and multivariate linear and non-linear models were explored, assessing superiority with Akaike Information Criterion (AIC).

## Results

**Fig. 2:**
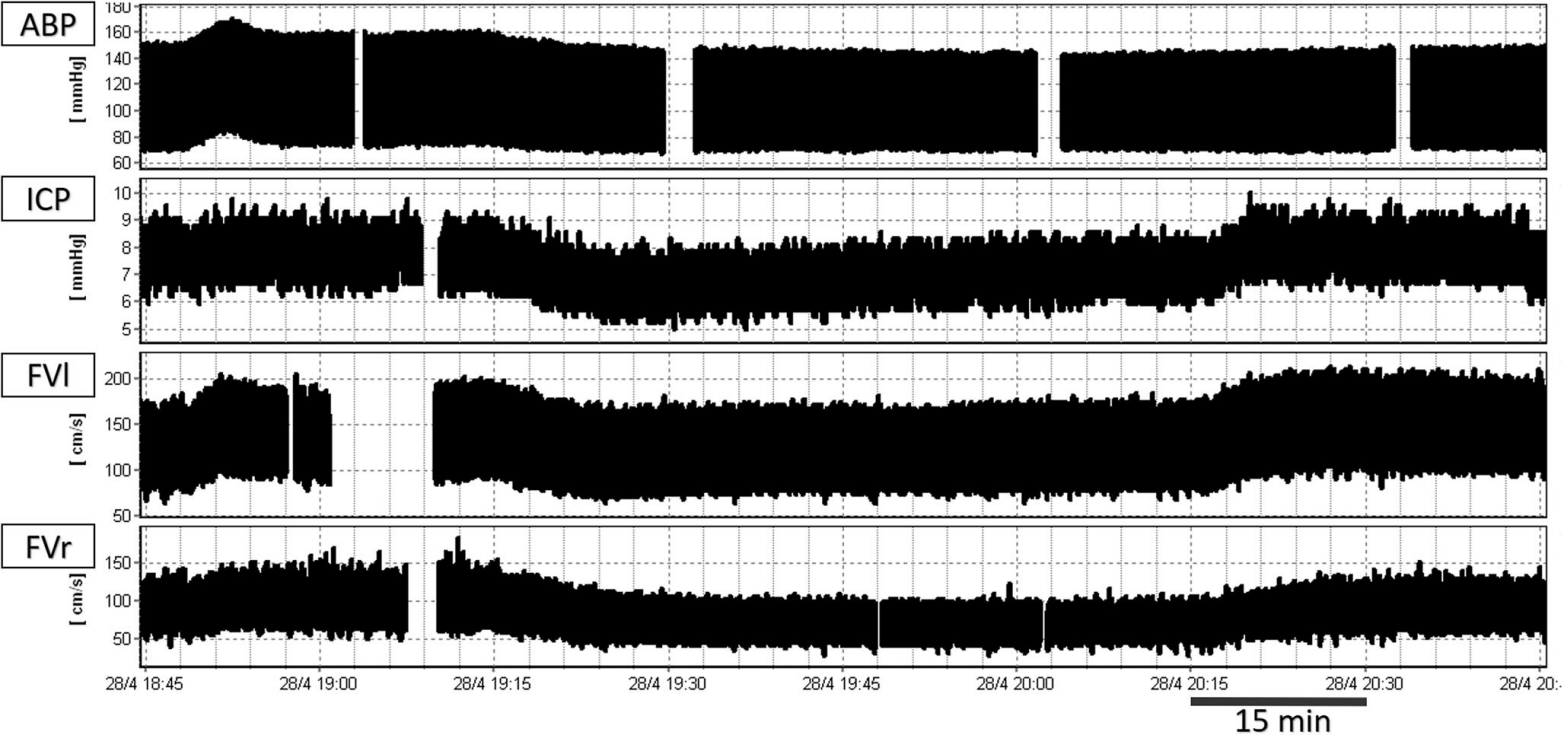
Example of the time trends of the recorded signals. Sampling frequency = 30 Hz. The gaps in the charts are due to the manual removal of artifacts such as arterial line flush, ICP transients, or FV disturbed signals. ABP, arterial blood pressure; ICP, intracranial pressure; FVl, flow velocity left; FVr, flow velocity right

**Fig. 3:**
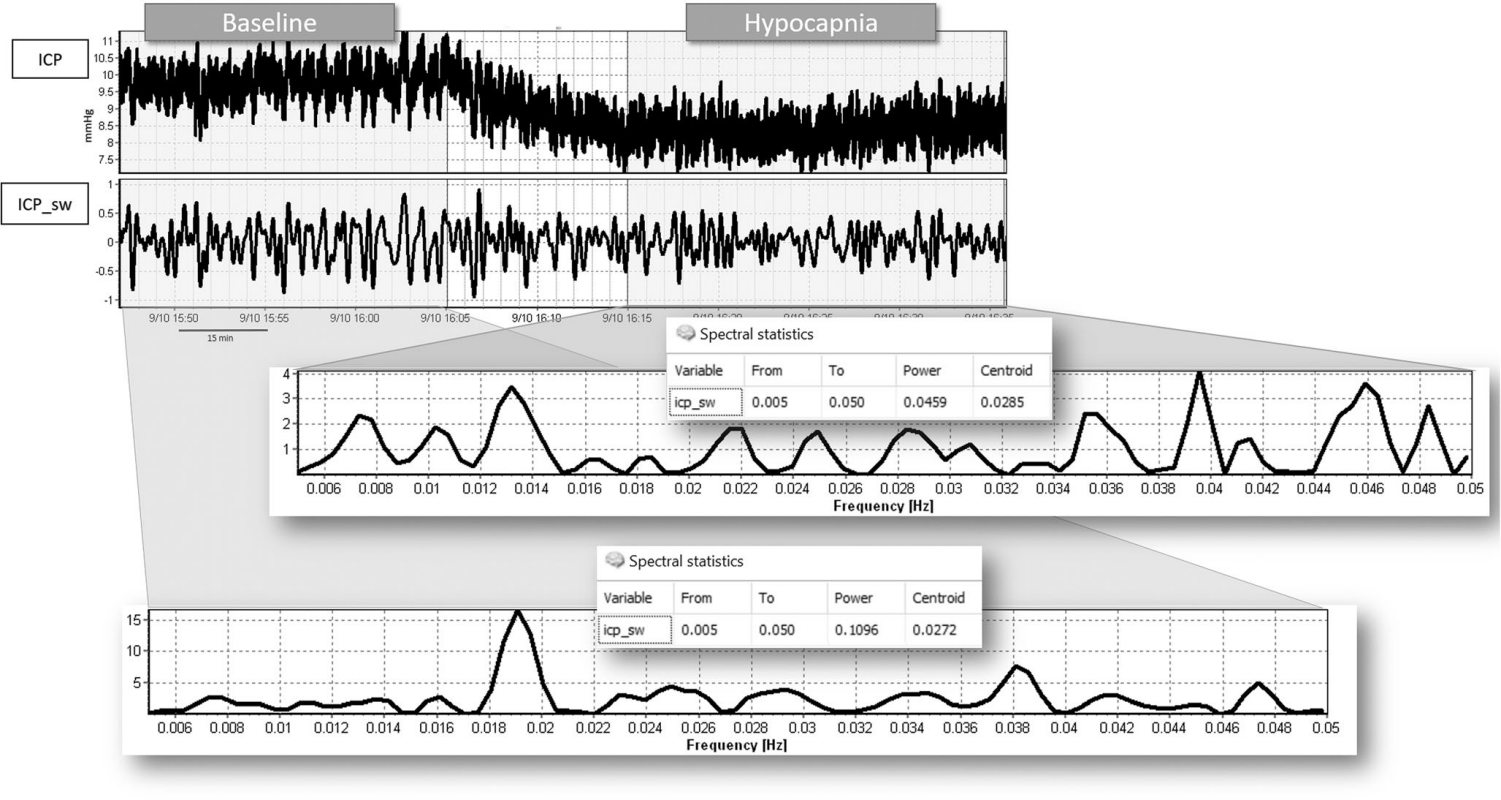
Time trend of the calculated ICP slow wave component and the PSD chart during the baseline and the hypocapnia period. ICP time series is presented as resampled to 1 Hz. ICP_sw shows the trend of the slow wave component of intracranial pressure as obtained by applying the FFT band-pass filter. Power spectral density analysis is shown (periodogram method using Hanning window) for the specific frequency range, for the two selected periods (baseline and hypocapnia). The correspondent power and centroid are shown in the spectral statistics table. ICP, intracranial pressure; ICP_sw, intracranial pressure slow waves time series; PSD, power spectral density

Twenty-nine adult (median age 39 years, 5 females) severe TBI patients (mean GCS 6 ± 3) were included. Figure 2 shows a typical example of the time trends of the recorded signals and Fig. 3 shows an example of the trend of the calculated slow waves component and the PSD chart during the baseline and the hypocapnia period, where mean pCO_2_ dropped from 5.10 ± 0.36 kPa to 4.39 ± 0.35 kPa, *p* < 0.001. Statistical comparisons of baseline versus hypocapnic period values are given in Table 1. Correlations between changes in ICP slow wave pattern and other physiological variables are presented in Table 2.

**Table 1:** Mean values and SD of the studied parameters at the baseline and during hypocapnia. When the changes between baseline and hypocapnia are significant, the direction of the change is highlighted. *p* values of univariate analysis are quoted uncorrected for multiple comparison

Variable			Baseline		Hypocapnia		Change	*p* value
			Mean	SD	Mean	SD		
Frequency domain	Power of sw (mmHg^2^)	ICP	1.04	1.82	0.27	0.49	↘	0.006*
		ABP	2.95	4.14	1.90	1.40		0.177
		FVl	16.02	20.48	10.90	13.66	↘	0.049*
		FVr	16.88	21.69	9.48	11.67	↘	0.005*
	Centroid of sw (Hz)	ICP	0.020	0.006	0.023	0.005	↗	< 0.001*
		ABP	0.016	0.005	0.020	0.006	↗	< 0.001*
		FVl	0.023	0.006	0.027	0.004	↗	< 0.001*
		FVr	0.022	0.006	0.026	0.005	↗	< 0.001*
Time domain, sw	Entropy of sw	ICP	0.50	0.11	0.56	0.06	↗	< 0.001*
		ABP	0.44	0.11	0.51	0.10	↗	< 0.001*
		FVl	0.55	0.07	0.58	0.05	↗	< 0.001*
		FVr	0.54	0.09	0.58	0.07	↗	< 0.001*
Time domain, time series	Time series (mmHg)	ICP	16.65	6.70	13.03	6.35	↘	< 0.001*
		ABP	96.45	8.98	98.83	11.65		0.100
		FVl	77.67	35.65	65.57	29.72	↘	< 0.001*
		FVr	78.14	26.82	61.65	17.50	↘	< 0.001*
	Coefficient of variation	ICP	0.09	0.05	0.10	0.08		0.206
		ABP	0.04	0.02	0.04	0.02		0.936
		FVl	0.07	0.03	0.08	0.04		0.071
		FVr	0.07	0.03	0.08	0.04		0.249
	Variance	ICP	2.23	2.16	1.39	1.39	↘	0.006*
		ABP	14.94	13.29	17.10	32.21		0.700
		FVl	37.10	36.29	39.37	71.75		0.825
		FVr	40.89	41.65	37.83	72.34		0.869
Time domain, derivate indexes	Coherence ICP-FVl		0.78	0.19	0.78	0.19		0.951
	Coherence ICP-FVr		0.76	0.21	0.76	0.19		0.401
	Ci_l (cm^3^/mmHg)		2.15	1.41	3.08	2.38	↗	0.006*
	Ci_r (cm^3^/mmHg)		2.36	1.63	3.07	2.69	↗	0.008*
	RAP		0.51	0.34	0.41	0.34	↘	0.026*

*sw* slow waves, *ICP* intracranial pressure, *ABP* arterial blood pressure, *FVl* flow velocity left, *FVr* flow velocity right, *Ci_l* compliance of cerebrospinal space left, *Ci_r* compliance of cerebrospinal fluid right, *RAP* compensatory reserve index

* denotes statistically significant finding

**Table 2:** Correlations between changes in ICP slow waves pattern and other physiological variables. The analysis is performed both for the changes between baseline and hypocapnia, and for the hypocapnic absolute values. The *p* values were not adjusted for multiple comparisons

	Variables	*r*	*p*
ICP-FV (baseline–hypocapnia)	∆*P*_ICP_; ∆*P*_FVl_	0.15	0.44
	∆*P*_ICP_; ∆*P*_FVr_	0.33	0.08
	∆*C*_ICP_; ∆*C*_FVl_	0.27	0.17
	∆*C*_ICP_; ∆*C*_FVr_	0.30	0.12
	∆*E*_ICP_; ∆*E*_FVl_	0.38	*0.04*
	∆*E*_ICP_; ∆*E*_FVr_	0.32	0.09
ICP-FV (during hypocapnia)	*P*_ICP_; *P*_FVl_	0.05	0.80
	*P*_ICP_; *P*_FVr_	0.06	0.74
	*C*_ICP_; *C*_FVl_	0.21	0.27
	*C*_ICP_; *C*_FVr_	0.40	*0.03*
	*E*_ICP_; *E*_FVl_	0.14	0.46
	*E*_ICP_; *E*_FVr_	0.31	0.10
ICP-Ci (during hypocapnia)	*P*_ICP_; Ci_l_	− 0.11	0.58
	*P*_ICP_; Ci_r_	− 0.14	0.47
ICP-Ci (baseline–hypocapnia)	∆*P*_ICP_; ∆Ci_l_	0.15	0.42
	∆*P*_ICP_; ∆Ci_r_	0.14	0.48
ICP-RAP	∆*P*_ICP_; ∆RAP	0.58	*0.001*
	∆*P*_ICP_; RAP_b_	0.71	*< 0.001*
FV-RAP	∆*P*_FVl_; ∆RAP	0.26	0.18
	∆*P*_FVr_; ∆RAP	0.50	*0.007*

∆ absolute delta value calculated as baseline–hypocapnia, *P* power of the slow waves, *E* entropy of the slow waves, *C* centroid of the slow waves, *ICP* intracranial pressure, *FVl* flow velocity left, *FVr* flow velocity right, *Cil* cerebrospinal compliance on the left side, *Cir* cerebrospinal compliance on the right side, *RAP* compensatory reserve index, *RAPb* compensatory reserve at the baseline

### Influence of hypocapnia on slow waves

According to the visual assessment of the time trends (Fig. 3), the slow wave component of ICP decreased in magnitude in 23/29 patients and did not change in 2/29.

The results of the statistical comparison baseline vs hypocapnia (Table 1) showed that this mainly affects ICP and FV (not ABP) and that they changed in the same direction in the following parameters: decrease in mean value (time series); decrease in power of slow waves; increase in centroid and therefore entropy of slow waves. ICP was the only variable with a significant change in variance of its mean value in the time domain. FVr-related parameters have a deeper decrease in hypocapnia if compared with the left side, except for the centroid and entropy of slow waves. The coefficient of variation did not change significantly, yet there was a tendency to increase during hypocapnia.

### Description of the correlation ICP-FV slow waves

The coherence between FV and ICP in the slow wave range was high (> 0.7) both at the baseline and during hypocapnia, with the left side coherence seemingly decreasing and the right side seemingly increasing during hypocapnia, but changes were not significant (Table 1). No statistical significance was found when ICP and FV slow waves–related metrics were correlated, except for changes in entropy at the left side and for absolute value of centroid on the right side (Table 2).

### Description of the correlation with compliance of brain CSF (Ci) space and compensatory reserve (RAP)

**Fig. 4:**
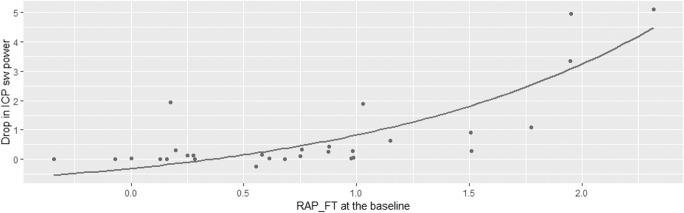
Relationship between RAP at the baseline (using the RAP fisher transformed) and the drop in power in ICP slow waves during hypocapnia (∆Power = Power_baseline_ − Power_hypocapnia_). This can be described as ∆*P*_ICP_ = − 1.3 + 2.1^RAPb^; adjusted *R* squared = 0.67. ICP, intracranial pressure; sw, slow waves; RAP_FT, compensatory index RAP after Fisher transformation

Ci increased significantly in the mean value from baseline to hypocapnia, while RAP decreased (Table 1). No statistical significance was found between changes in ICP slow waves and Ci (Table 2). RAP_FT was used for the following correlations (Table 2). The delta power of ICP slow waves was correlated to the delta RAP and so did the mean value of RAP at the baseline with even stronger correlation (Fig. 4). Delta RAP at the baseline was not correlated with the delta power of FV slow waves on the left side, but it was on the right side.

A multiple linear regression model incorporating all the physiological variables (RAP at the baseline (RAP_b_), delta Ci and delta power of FV slow waves) was investigated in order to identify factors that could better predict the decrease in the power of ICP slow waves. The results suggested RAP at the baseline (*p* < 0.001) as the strongest predictor for the decrease in the power of ICP slow waves (∆*P*_ICP_). Other, reduced, models were compared with the full one, and the superiority was assessed with AIC. If only RAP_b_ was considered in the model (non-linear), ∆*P*_ICP_ could be described as

$$ \Delta  {P}_{\mathrm{ICP}}=-1.3+{2.1}^{\mathrm{RAPb}} $$

This showed the best fit according to the AIC test and expressed 67% of the total variation of the drop in power of ICP slow waves (Fig. 4).

## Discussion

In this study, we intended to scrutinize the behavior of the slow waves of ICP during short hypocapnia tests in TBI patients and to relate it to the other relevant physiological variables.

Given the physiological information carried by the ICP slow waves, several different methods have been proposed for their qualitative and quantitative analysis. Eklund et al. [11] described two computerized methods, one in the time domain (waveform analysis) and one in the frequency domain (estimation of B waves power in 10 min blocks of ICP monitoring). Hara et al. [13] used an automated offline detection of slow waves based on the power spectrum of ICP oscillations by fast Fourier transform (FFT), while Walter et al. [38] proposed an online version based on an ARMA modeling derived spectral estimation. Kasprowicz et al. [18] focused on the analysis of the morphology changes of individual ICP pulses during the slow waves. Spiegelberg et al. [34] applied a pattern recognition in the time domain. However, there is not any apparent advantage of using one method over another and the description of the changes in slow waves in different conditions depends on the methods utilized. We therefore firstly defined a methodology that allowed us to approach the slow waves both in the time and frequency domains and to describe them in terms of total power, frequencies patterns, and time domain variability in our dataset. We defined the slow waves in terms of their frequency, range of 0.005 to 0.05 Hz, not specifying a minimum value for their amplitude.

As expected from the visual inspection, the spectral power of the slow waves decreased significantly in ICP and in FV during hypocapnia when compared with the baseline period. However, the range of frequencies where the power was concentrated was widened with a shift of the center of gravity toward the higher frequencies (an increase of the centroid of the slow waves). Overall, if the activity of the slow waves is defined only by the total power, then we can state that hypocapnia decreases the slow waves activity. But, if we consider the frequency distribution characteristics as part of the slow waves activity signature, then this is perhaps not entirely true anymore, with the centroid change indicating increase in the higher frequency content of the waves (more “complex” waveform morphology), counterbalancing, in a way, the power decrease.

The supposition of a more complex waveform pattern during hypocapnia seems to be confirmed by the observed increase in the sample entropy calculated in the time domain of the slow waves. The meaning of this needs to be clarified. In biological systems, higher entropy of the relevant physiological measurement time series usually indicates a healthier system, while a breakdown in homeostasis usually leads to its decrease [12]^-^[28]^-^[33]. In our dataset, suddenly induced mild-moderate hypocapnia increased the entropy related to the slow waves, suggesting a potential improvement in the regulatory capacity of the whole cerebrovascular system.

The power of ABP slow waves did not seem to be affected by hypocapnia since the decrease did not reach a statistical significance. This suggests that the observed changes in ICP and FV slow waves pattern were either due to changes in the transmission of the ABP waves to CBV, or that they were caused by an independent, intracranial, cerebrovascular tone modulating mechanism affected by hypocapnia. However, the fact that the frequency composition/waveform morphology showed the same pattern of change as the other two modalities (as indicated by the centroid and entropy metrics) seems to favor the former interpretation.

**Fig. 5:**
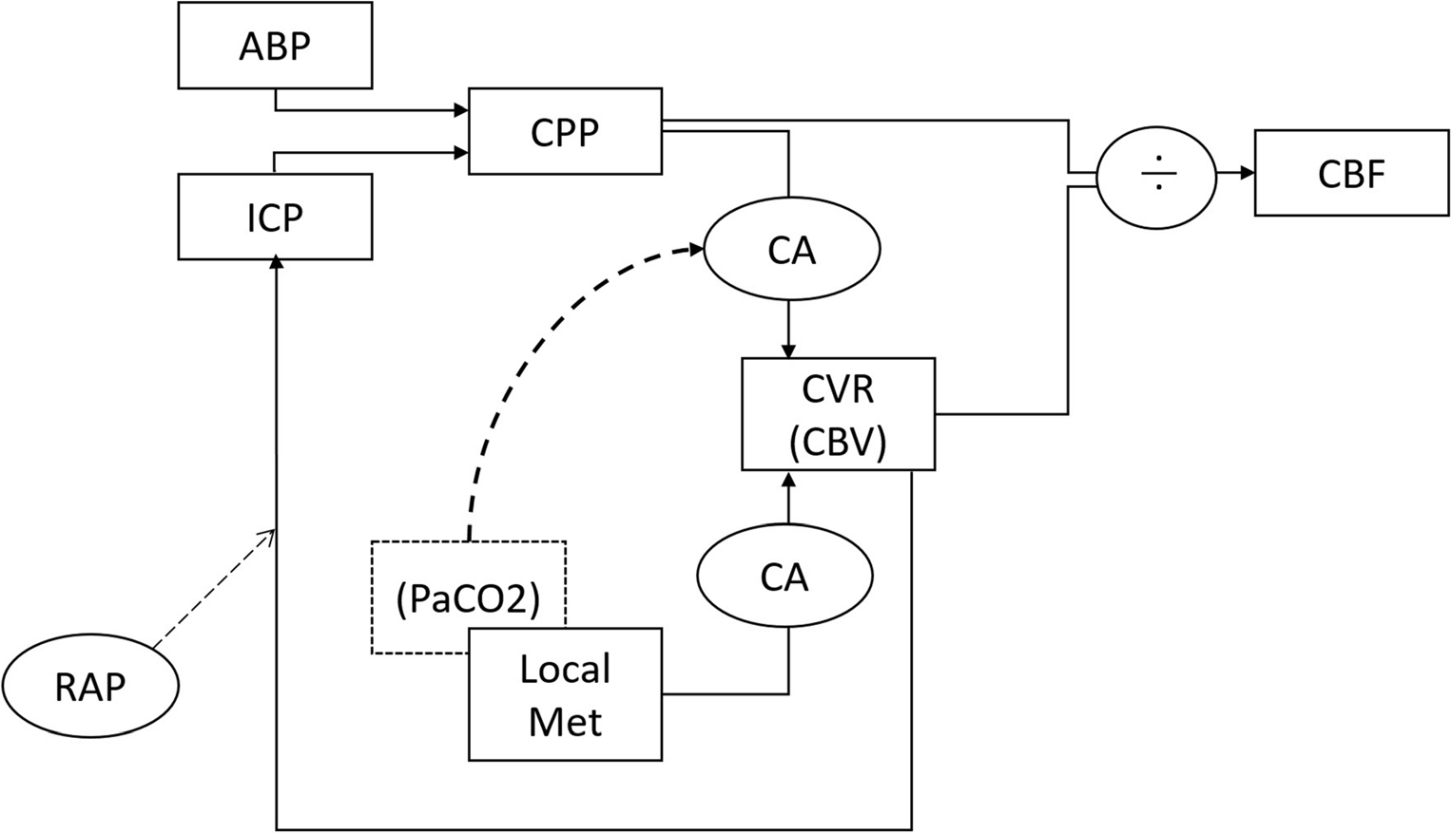
Mechanisms involved in the generation and transmission of the slow waves in the intracranial vault. ABP, arterial blood pressure; ICP, intracranial pressure; CPP, cerebral perfusion pressure; CA, cerebral autoregulation; CVR, cerebrovascular resistance, CBV, cerebral blood volume; Met, metabolic factors; PaCO2, arterial carbon dioxide pressure; RAP, compensatory reserve index; CBF, cerebral blood flow

Induced hypocapnia causes, with intact cerebrovascular reactivity, a cerebral vasoconstriction, which ultimately leads to a decrease in CBV and thus ICP. As cerebrovascular resistance increases, a decrease in FV will follow if significant changes in ABP do not occur (Fig. 5). The vasogenic waves generated by the dynamic changes in CBV at the two vascular diameter levels (relatively dilated baseline and relatively constricted hypocapnia) will have different amplitude depending on the vascular tone (a fall in the vascular tone causes an increase in the amplitude of the vascular waves) [1]. A parameter called vascular “wall tension” (WT) is considered an indicator of the arterial smooth muscles tones. This parameter has been investigated in the same data set by Smielewski et al. in a previous study: WT was shown to increase during hypocapnia, leading to a stiffer arterial bed and thus attenuated transmission of arterial pressure waves. In the same study, the cerebrovascular reactivity was shown to be intact for both levels of CO2, with no significant difference between the two [32].

We further considered the transmission of the generated vasogenic slow waves into FV [25] and in ICP waveforms [1]. FV and ICP slow waves activity shows a similar pattern change after the suddenly induced hypocapnia if compared with the preceding baseline period (decreased power and increased centroid frequency), which might be explained by the common origin of the vasogenic waves. However, we did not find a significant correlation between the slow waves related metrics in ICP and FV. The power modification in the FV slow waves might be explained by the fact that ABP slow waves power also showed a tendency to decrease during hypocapnia, even if that did not quite reach statistical significance. Clearly, a different mechanism from the variation in the CBV fluctuations must be responsible for the modification in the ICP slow waves pattern during hypocapnia.

What changes here is probably the “transmission factor,” related to cerebrospinal compliance and compensatory reserve (Fig. 5). The amplitude of the ICP slow waves generally increases in low compensatory reserve [39] and Lundeberg postulated their rise in low compliance [24]. Given that a decrease in CBV causes an improvement in the compliance (which we also observed), we investigated the relationship between cerebral compliance and the hypocapnia induced variation in the ICP slow waves pattern.

We did not find a correlation between the variation in total power of ICP slow waves and the Ci, which suggests that the CSF and venous compartment are not the only ones playing a role in the decrease in the power of ICP slow waves. Interestingly, the drop in power of the ICP slow waves was correlated to the increase in compensatory reserve from baseline to hypocapnia and more strongly to the baseline compensatory reserve. RAP is a more comprehensive index then Ci, because it takes into account the multiple cerebral compartments. The worse the compensatory reserve at the baseline, meaning the tighter the brain before the hypocapnic challenge, the higher the difference in the power of ICP slow waves induced by hypocapnia. We suggest here a non-linear model describing this relationship (Fig. 4), where variation in the power of ICP slow waves increases exponentially with RAP at the baseline. Similarly, Steiner et al. [35] showed in the same dataset that RAP at the baseline was the strongest predictor of the reduction of ICP mean value during hypocapnia.

In TBI patients, therefore, induced mild to moderate hypocapnia provokes on the one hand a decrease in ICP slow waves power (the magnitude of which depends on the compensatory reserve during the previous normocapnia period) and on the other hand an increase of the ICP slow wave centroid (increased higher frequencies contribution) and improvement of their entropy. Quantifying these relationships may be clinically relevant when the metrics derived from the slow waves analysis, such as cerebral autoregulation (via assessment of the pressure reactivity index PRx) [36], brain stem activity [22],[15], and cerebral compliance [24, 27], are used in daily monitoring and integrated with the other clinical diagnostic “tools.” The change in power may need to be correlated to the state of the compensatory reserve and perhaps should be taken into account when interpreting the derived metrics, given the correlation with the power of slow waves and clinical outcome. The variability induced in the morphological content on the other hand could provide further insights into the cerebrovascular system, confirming the non-harmful effect of short term hypocapnic challenges used for treating intracranial hypertension.

## Limitations

In this retrospective study, only 29 patients were investigated and the patient heterogeneity as well as the injury pattern heterogeneity was not taken into account. Further studies with a larger cohort of patients will be needed to validate these preliminary findings.

Although the ventilator settings were kept constant during the hypocapnia challenge and during the period directly preceding it, we cannot exclude small, but possibly influential, variations in PaCO_2_. Having EtCO_2_ measurements available with our data set would go some way toward further reassurance of stable PaCO_2_ levels.

The hypocapnic periods were selected in the very first hypocapnic period after the stabilization; therefore, our findings might not reflect what happens in late hypocapnia. Further investigations are required to describe the behavior of intracranial slow waves in late hypocapnia.

In the same way, deep hypocapnia may show different results vs mild hypocapnia. Therefore, changes in slow waves during deep hypocapnia should be studied separately.

From the methodological point of view, the artifacts and in particular the “transient” patterns were defined heuristically by the investigators. However, the choice was kept consistent. For future studies, an agreement on the artifacts and transients definition should be achieved.

Moreover, even if the description of the pattern of the slow waves during the immediate post-hypocapnia period would have been desirable and informative, a clear post-hypocapnia period could not be identified in a reliable way and a comparison hypocapnia vs post-hypocapnia state was not possible.

## Conclusions

We found that in severe TBI patients, a sudden mild to moderate hypocapnia induces a decrease in ICP and FV slow wave power. It also increases their higher frequency content and their morphological complexity (entropy). The difference in power of the ICP slow waves between the baseline and the hypocapnia period depends on the baseline compensatory reserve, as expressed by RAP index.

## Abbreviations and acronyms

TBI: Traumatic brain injury
ICP: Intracranial pressure
ABP: Arterial blood pressure
FV: Flow velocity
Ci: Cerebrospinal compliance
FFT: Fast Fourier transform
RAP: Compensatory reserve index
CBF: Cerebral blood flow
CBV: Cerebral blood volume
MCA: Middle cerebral artery
CPP: Cerebral perfusion pressure
PaCO2: Arterial partial pressure of carbon dioxide
pCO_2_: Partial pressure of carbon dioxide
FVl: Flow velocity left
FVr: Flow velocity right
SjvO_2_: Jugular bulb venous oxygen saturation
sw: Slow wave
RAP_FT: Fisher transformation of RAP
PSD: Power spectral density
ABP_sw: Slow wave component of ABP
ICP_sw: Slow wave component of ICP
FVl_sw: Slow wave component of FVl
FVr_sw: Slow wave component of FVr
CaBV: Cerebral arterial blood volume

## References

[1] Auer LM, Sayama I (1983). Intracranial pressure oscillations (B-waves) caused by oscillations in cerebrovascular volume. Acta Neurochir.

[2] Balestreri M, Czosnyka M, Steiner LA, Schmidt E, Smielewski P, Matta B, Pickard JD (2004). Intracranial hypertension: what additional information can be derived from ICP waveform after head injury?. Acta Neurochir.

[3] Brady KM, Easley RB, Kibler K (2012). Positive end-expiratory pressure oscillation facilitates brain vascular reactivity monitoring. J Appl Physiol.

[4] Brian JE (1998). Carbon dioxide and the cerebral circulation. Anesthesiology.

[5] Carrera E, Steiner LA, Castellani G, Smielewski P, Zweifel C, Haubrich C, Pickard JD, Menon DK, Czosnyka M (2011). Changes in cerebral compartmental compliances during mild hypocapnia in patients with traumatic brain injury. J Neurotrauma.

[6] Coles JP, Fryer TD, Coleman MR (2007). Hyperventilation following head injury: effect on ischemic burden and cerebral oxidative metabolism. Crit Care Med.

[7] Czosnyka M, Pickard JD (2004). Monitoring and interpretation of intracranial pressure. J Neurol Neurosurg Psychiatry.

[8] Czosnyka M, Price DJ, Williamson M (1994). Monitoring of cerebrospinal dynamics using continuous analysis of intracranial pressure and cerebral perfusion pressure in head injury. Acta Neurochir.

[9] Czosnyka M, Smielewski P, Kirkpatrick P, Laing RJ, Menon D, Pickard JD (1997). Continuous assessment of the cerebral vasomotor reactivity in head injury. Neurosurgery.

[10] Einhäupl KM, Garner C, Dirnagl U, Schmieder G, Schmiedek P, Kufner G, Rieder J (1986) Oscillations of ICP related to cardiovascular parameters BT - intracranial pressure VI. In: Miller JD, Teasdale GM, Rowan JO, Galbraith SL, Mendelow AD (eds). Springer Berlin Heidelberg, Berlin, Heidelberg, pp 290–297

[11] Eklund A, Agren-Wilsson A, Andersson N, Bergenheim AT, Koskinen LO, Malm J (2001). Two computerized methods used to analyze intracranial pressure B waves: comparison with traditional visual interpretation. J Neurosurg.

[12] Goldstein B, Fiser DH, Kelly MM, Mickelsen D, Ruttimann U, Pollack MM (1998). Decomplexification in critical illness and injury: relationship between heart rate variability, severity of illness, and outcome. Crit Care Med.

[13] Hara Keita, Nakatani Susumu, Ozaki Kohji, Mogami Heitarou, Ikeda Takuya (1990). Detection of the B waves in the oscillation of intracranial pressure by fast Fourier transform. Medical Informatics.

[14] Harper AM (1966). Autoregulation of cerebral blood flow: influence of the arterial blood pressure on the blood flow through the cerebral cortex. J Neurol Neurosurg Psychiatry.

[15] Higashi S, Yamamoto S, Hashimoto M, Fujii H, Ito H, Kogure Y, Tokuda K (1989) The role of vasomotor center and adrenergic pathway in B-waves BT - intracranial pressure VII. In: Hoff JT, Betz AL (eds). Springer, Berlin Heidelberg, Berlin, Heidelberg, pp 220–224

[16] Hundley WG, Renaldo GJ, Levasseur JE, Kontos HA (1988). Vasomotion in cerebral microcirculation of awake rabbits. Am J Physiol.

[17] Jones SC, Williams JL, Shea M, Easley KA, Wei D (1995). Cortical cerebral blood flow cycling: anesthesia and arterial blood pressure. Am J Physiol.

[18] Kasprowicz M, Asgari S, Bergsneider M, Czosnyka M, Hamilton R, Hu X (2010). Pattern recognition of overnight intracranial pressure slow waves using morphological features of intracranial pressure pulse. J Neurosci Methods.

[19] Kay SM (1988) Modern spectral estimation: theory and application. Prentice Hall

[20] Kuo TB-J, Chern C-M, Sheng W-Y, Wong W-J, Hu H-H (1998). Frequency domain analysis of cerebral blood flow velocity and its correlation with arterial blood pressure. J Cereb Blood Flow Metab.

[21] Lalou DA, Czosnyka M, Donnelly J, Lavinio A, Pickard JD, Garnett M, Czosnyka Z (2016). Influence of general anaesthesia on slow waves of intracranial pressure. Neurol Res.

[22] Lang EW, Diehl RR, Timmermann L, Baron R, Deuschl G, Mehdorn HM, Zunker P (1999). Spontaneous oscillations of arterial blood pressure, cerebral and peripheral blood flow in healthy and comatose subjects. Neurol Res.

[23] Lu C-W, Czosnyka M, Shieh J-S, Pickard JD, Smielewski P (2016). Acta Neurochir Suppl.

[24] Lundberg N (1960). Continuous recording and control of ventricular fluid pressure in neurosurgical practice. Acta Psychiatr Scand Suppl.

[25] Mautner-Huppert D, Haberl RL, Dirnagl U, Villringer A, Schmiedek P, Einhäupl K (1989). B-waves in healthy persons. Neurol Res.

[26] Muizelaar JP, Marmarou A, Ward JD, Kontos HA, Choi SC, Becker DP, Gruemer H, Young HF (1991). Adverse effects of prolonged hyperventilation in patients with severe head injury: a randomized clinical trial. J Neurosurg.

[27] Newell DW, Aaslid R, Stooss R, Reulen HJ (1992). The relationship of blood flow velocity fluctuations to intracranial pressure B waves. J Neurosurg.

[28] Norris PR, Anderson SM, Jenkins JM, Williams AE, Morris JA (2008). Heart rate multiscale entropy at three hours predicts hospital mortality in 3,154 trauma patients. Shock (Augusta, Ga).

[29] Richman JS, Moorman JR (2000). Physiological time-series analysis using approximate entropy and sample entropy. Am J Physiol Heart Circ Physiol.

[30] Rosner MJ, Miller JD, Teasdale GM, Rowan JO, Galbraith SL, Mendelow AD (1986). The vasodilatory cascade and intracranial pressure. Intracranial Pressure VI.

[31] Russo G, Lodi CA, Ursino M (2000). Quantitative assessment of cerebral vascular reserve by means of transcranial Doppler ultrasound and rebreathing maneuver: bedside test and mathematical modeling. Neurol Sci.

[32] Smielewski P, Steiner LA, Puppo C, Budohoski K, Varsos GV, Czosnyka M (2018) Effect of mild hypocapnia on critical closing pressure and other mechanoelastic parameters of the cerebrospinal system. Acta Neurochir Suppl 126:139–142 10.1007/978-3-319-65798-1_29 29492549

[33] Sortica da Costa C, Placek MM, Czosnyka M, Cabella B, Kasprowicz M, Austin T, Smielewski P (2017). Complexity of brain signals is associated with outcome in preterm infants. J Cereb Blood Flow Metab.

[34] Spiegelberg A, Krause M, Meixensberger J, Seifert B, Kurtcuoglu V (2018) Significant association of slow vasogenic ICP waves with normal pressure hydrocephalus diagnosis. Acta Neurochir Suppl:243–246 10.1007/978-3-319-65798-1_49 29492569

[35] Steiner LA, Balestreri M, Johnston AJ, Coles JP, Smielewski P, Pickard JD, Menon DK, Czosnyka M (2005). Predicting the response of intracranial pressure to moderate hyperventilation. Acta Neurochir.

[36] Steinmer R, Rauhuf C, Hübner U, Bauer R, Fahlbusch R, Laumer R, Bondar I (1996). Slow rhythmic oscillations of blood pressure, intracranial pressure, microcirculation, and cerebral oxygenation. Dynamic interrelation and time course in humans. Stroke.

[37] Venes JL (1979). B waves – a reflection of cardiorespiratory or cerebral nervous systems rhythm?. Pediatr Neurosurg.

[38] Walter M, Kiefer M, Leonhardt S, Steudel WI, Isermann R (2002). Online analysis of intracranial pressure waves. Acta Neurochir Suppl.

[39] Weerakkody RA, Czosnyka M, Zweifel C, Castellani G, Smielewski P, Keong N, Haubrich C, Pickard J, Czosnyka Z (2010). Slow vasogenic fluctuations of intracranial pressure and cerebral near infrared spectroscopy--an observational study. Acta Neurochir.

[40] Yoshihara M, Bandoh K, Marmarou A (1995). Cerebrovascular carbon dioxide reactivity assessed by intracranial pressure dynamics in severely head injured patients. J Neurosurg.

